# Psychological distress and quality of life following provision of vascular imaging results of the coronary and carotid arteries to asymptomatic adults: a scoping review protocol

**DOI:** 10.12688/f1000research.27432.2

**Published:** 2021-05-28

**Authors:** Reindolf Anokye, Ben Jackson, James Dimmock, Joanne M. Dickson, Lauren C. Blekkenhorst, Jonathan M. Hodgson, Joshua R. Lewis, Mandy Stanley

**Affiliations:** 1 School of Medical and Health Sciences, Edith Cowan University, Joondalup, Western Australia, 6027, Australia; 2 School of Human Sciences (Exercise and Sport Science), University of Western Australia, Crawley, Western Australia, 6009, Australia; 3 Telethon Kids Institute, Perth, Western Australia, Australia; 4 Department of Psychology, James Cook University, Townsville, Queensland, 4811, Australia; 5 School of Arts and Humanities (Psychology Discipline), Edith Cowan University, Joondalup, Western Australia, 6027, Australia; 6 Department of Psychological Science, University of Liverpool, Liverpool, L69 7ZX, UK; 7 Medical School, University of Western Australia, Crawley, Western Australia, 6009, Australia; 8 School of Public Health, Sydney Medical School, The University of Sydney, Sydney, New South Wales, 2000, Australia

**Keywords:** Mental distress, Quality of life, Non-invasive vascular imaging, Asymptomatic adults, Scoping review

## Abstract

**Background: **Non-invasive screening for atherosclerosis or asymptomatic cardiovascular disease of the coronary and carotid arteries is commonly undertaken, and research has been focussed on how results from these screenings lead to behaviour change. However, no review has focused on the effects of these results on psychological distress and health-related quality of life (HRQoL). This protocol will outline how a scoping review will be conducted to map all available evidence on psychological distress and/or HRQoL outcomes following the provision of vascular imaging results of the coronary and carotid arteries.

**Methods: **Arksey and O’Malley’s (2005) framework subsequently enhanced by Levac et al. (2010)  and Peters et al (2015, 2017) will guide the scoping review. Databases such as MEDLINE (Clarivate), APA PsychINFO, EMBASE, Social Work Abstracts, Psychology and Behavioural Sciences Collection, and Cumulative Index to Nursing and Allied Health Literature (CINAHL) will be searched using MeSH terms  such as "Coronary stenosis", "Carotid Stenosis", "Psychological Distress" and "Quality of Life" and related terms. Two investigators will screen title and abstract and all articles meeting inclusion criteria will be extracted.  Data on authors, publication year, country of origin, aims/purpose, methodology, intervention, outcome measures as well as key findings that relate to the scoping review questions will be extracted for each included study. The findings will be presented using tables and thematic narrative synthesis. The scoping review will not produce a pooled estimate of the impact of vascular imaging results on psychological distress and HRQoL but will present information from the included studies related to psychological distress and HRQoL.

**Conclusion:**  The review will highlight and address gaps in knowledge and provide direction for future investigations.

## Introduction

Cardiovascular disease (CVD) refers to diseases of the blood vessels, and in particular the heart, brain and peripheral vasculature ^1^. CVDs due to atherosclerosis include cerebrovascular events such as stroke, ischaemic heart disease events such as heart attacks, and peripheral arterial diseases causing peripheral claudication ^1^. CVD is the leading cause of death and disability globally ^1, 2^ with an estimated 17.9 million people dying from CVDs in 2016, representing 31% of all global deaths. Of these CVD-related deaths, 85% were due to heart attack and stroke or their sequelae ^2^. By 2030, it is estimated that more than 22.2 million people will die annually from CVDs ^3^.

Atherosclerosis before clinical events, or “asymptomatic CVD”, can be easily visualised using a range of imaging methods, with the most common being computed tomography of the coronary arteries to calculate coronary artery calcification (CAC) or carotid ultrasound to identify carotid plaques and assess intimal medial thickness ^4^. Imaging of the arteries to identify asymptomatic CVD is becoming commonplace in medical practice ^5^, and provides asymptomatic individuals with a visible and tangible illustration of an otherwise hidden disease process, even before distinctive symptoms appear ^6^. Such information can improve an individual’s knowledge of the disease which may enable them to increase control over, and improve their health ^7^. Increased knowledge may also lead to personal and social benefits, such as enabling effective community action and contributing to developing one's social capital ^8, 9^. However, diagnostic information or results related to a disease (depending on how the situation is evaluated) may also affect an individual’s sense of well-being ^10^ or lead to psychological distress ^6, 11, 12^. For example, previous studies have reported that women who undergo mammography screening may be susceptible to psychological distress following the provision of results ^13– 21^.

Psychological distress, often referred to as mental distress, refers to the unique discomforting emotional state an individual experiences in response to a particular demand or stressor that causes temporary or permanent harm to them ^22^. Psychological distress often manifests through attributes including: (a) discomfort (e.g. anguish, misery, suffering); (b) perceived inability to effectively cope (e.g. inability to solve problems); (c) communication of discomfort (e.g. facial expressions); (d) loss of independence and confidence (e.g. dependency, decreased self-esteem);and/or (e) changes in emotional status (e.g. change from stable emotional state to one of depression, self-depreciation, amotivation, dysregulated motivation or maladaptive motivation,aggressiveness, irritability, nervousness, and anxiety) ^23– 33^. Quality of Life (QOL) encompasses a person’s psychological state, appraisals of physical health, personal beliefs as well as social relationships ^34^. It is often measured in research using physical and mental health summary scores ^35^. This review focuses on health-related quality of life (HRQoL), which refers to a multidimensional construct encompassing appraisals of physical and emotional health, wellness or illness ^36– 38^. HRQoL is generally considered as the most suitable variant of quality of life when one is investigating medical conditions or disease related outcomes ^39^. HRQoL and psychological distress have been extensively studied in health research ^40– 46^. Reported impaired HRQoL (e.g. illness , role limitations due to physical or emotional/psychological problems), improved HRQoL (e.g. wellness, improved physical or mental health) and psychological distress (e.g. anxiety, depression, worry) following screening are the outcomes of interest for this scoping review. Psychological outcomes will be reported under HRQoL domain in this scoping review if they were categorised as QoL/HRQoL in the included studies (e.g., measured using validated QoL/HRQoL instrument and reported as impaired/improved QoL/HRQoL). Psychological outcomes will also be reported under psychological distress domain in this scoping review if they were measured and reported as a single psychosocial variable (e.g., depression, self-esteem, anxiety).

The scoping review was informed by Witte’s ^47^ extended parallel process model (EPPM) and cognitive stress appraisal theory ^48^. Based on the constructs of the EPPM ^47^, the provision of information—in particular, negative information—about a person’s coronary artery calcium and carotid plaque (and the potential implications of this condition) is likely to stimulate subjective ‘threat’ appraisals (i.e., perceived susceptibility to, and severity of, CVD) ^49^. Depending on interactions between that threat appraisal and individuals’ efficacy appraisals, individuals may react to screening information by (a) adopting danger control responses (including attitudes, beliefs, behavioural intentions, and/or behaviours) that align with message recommendations, or (b) adopting fear control processes (such as denial, reactance, and avoidance) intended to reduce fear rather than take protective action ^50^. Behavioural intentions and/or behaviours such as increasing physical activity, health responsibility, good nutrition, and stress management could impact health outcomes ^51^. Behavioural intentions and/or behaviours are also associated with lifestyle related disease burden such as CVD ^52^ which could undermine HRQoL ^53^. Cognitive stress appraisal theory ^48^ also proposes that individuals primarily evaluate circumstances/situations as ‘challenging’ (i.e., threat that can be overcome or met) or ‘threatening’ (i.e., anticipated loss/harm) ^48^. Positive cognitive stress appraisal (i.e. appraising a situation as a challenge to be resolved and setting goals to achieve that) may contribute to prevention of depression and improved HRQoL ^54^. Negative appraisals of stress—viewing an issue such as detected atherosclerotic plaque in the arteries as a threat and believing that resolving it is beyond one’s abilities—may, however, lead to psychological distress ^55– 57^.

Based on the EPPM and cognitive stress appraisal frameworks, we therefore hypothesized that; (a) population screening to detect atherosclerotic plaque in the coronary or carotid arteries can influence HRQoL, and (b) population screening to detect atherosclerotic plaque in the coronary or carotid arteries can cause psychological distress. To date, however, the available evidence that may support (or refute) these hypotheses has not been scrutinised or reported in any coherent manner. Hence, there is a need for a scoping review to synthesize the state of scientific literature on this subject.

Scoping reviews aim to map key concepts, main sources and types of evidence available in a research area and can be undertaken where an area is complex or has not been comprehensively reviewed before ^58^. Previous reviews reported very little evidence relating to HRQoL or psychological distress following provision of vascular imaging results to asymptomatic adults ^7, 59– 62^. It is important, therefore, to collate evidence relating to the findings available in this field, how studies in this field have been conducted, the key characteristics of studies, and important knowledge gaps. As such, this scoping review will comprehensively map the evidence on psychological distress and HRQoL outcomes following provision of vascular imaging results of the coronary or carotid arteries to asymptomatic adults. We will also report other details of included studies that we deem important in this scoping review (e.g. the information provided during counselling and whether the counselling could reduce distress or any information included in the results that shaped the nature of the response).

## Study rationale and guiding question

There is great interest (and value) in providing people with vascular imaging results of the coronary and carotid arteries to prompt healthful behaviour change and better management of CVD ^7, 60^. However, the provision of the imaging results may produce markedly different emotions—and as a result, downstream behaviours—depending upon the way in which they are received and appraised. Also, the uncertainty about a possible future threat (due to coronary artery calcium and carotid plaque) may cause anxiety ^63^. There is theoretical justification to anticipate that information aimed at prompting healthful behaviour change and better management of CVD may stimulate negative psychosocial outcomes or psychological distress such as anxiety or depression impairing HRQoL. Accordingly, it is important to identify which research questions have and have not been addressed in this area. Also, by highlighting the extent of findings on distress and/or HRQoL, a scoping review could support the development of strategies designed to mitigate or prevent distress during and following such screening exercises.

The aim of this review is to map all available evidence on psychological distress and HRQoL outcomes among participants who were screened for atherosclerosis by non-invasive methods and provided with their own coronary or carotid artery vascular imaging results. This scoping review will address this research question:

1. What is the state of scientific literature on psychological distress and HRQoL related to the provision of vascular imaging results of the coronary and carotid arteries, and what are the gaps in that literature?

Table 1 further clarifies the core elements of the questions guiding the conduct of this scoping review.

**Table 1.** An overview of core elements of scoping review questions. HRQoL, health-related quality of life.

CORE ELEMENTS	EXPLANATION
SCOPE OF THE REVIEW	Global
SETTING	Community and/or clinical settings
POPULATION	Adult participants who have been screened for coronary artery calcium/calcification or carotid plaque/ stenosis
INTERVENTION	Screening for atherosclerosis in the coronary or carotid arteries using non-invasive imaging techniques
COMPARISON	1. Reported psychological distress and/or HRQoL in sub-groups provided with results of detected atherosclerotic plaque after screening and those without 2. Reported psychological distress and/or HRQoL in sub-groups within different risk categories (e.g. no risk/normal, low risk, mild risk, moderate risk and high risk groups) 3. Reported psychological distress and/or HRQoL in sub-groups with knowledge of test results and those without 4. Reported psychological distress and/or HRQoL in sub-groups screened and provided results and non- screening group 5. Reported psychological distress and/or HRQoL in populations before and after provision of results
EVALUATION	Reported changes/no changes or differences/no differences in psychological distress and/or HRQoL following the provision of vascular imaging results of an individual’s carotid or coronary arteries; how studies were conducted and important knowledge gaps.

## Protocol

### Methods

***Study design***. The framework initially developed by Arksey and O’Malley ^64^ and subsequently enhanced by Levac *et al.* ^65^ and Peters *et al.* ^66– 68^ will be used for this scoping review. The framework involves stages such as: (1) identifying, clarifying, defining and linking the purpose of the study and the research question; (2) identifying relevant studies, balancing comprehensiveness and breadth with feasibility; (3) developing and aligning inclusion criteria with study questions and objectives; (4) using an iterative approach to study selection and data extraction; (5) using a planned approach to searching evidence, study selection, extracting data, and evidence presentation; (6) incorporating qualitative thematic analysis and numerical summary to collating, summarizing and reporting the results; and (7) Summarizing the evidence in relation to the aims of the review, making conclusions and identifying any implications for practice, policy or research. The reporting of this scoping review will also be guided by the PRISMA extension for scoping review reporting checklist ^69^.

### Identifying relevant studies

***Information sources and search strategy*.** The main purpose of a scoping review is to comprehensively identify primary studies (published and unpublished) and reviews suitable for answering the review questions. To achieve this, databases such as MEDLINE (Clarivate), APA PsychINFO, EMBASE, Social Work Abstracts, Psychology and Behavioural Sciences Collection, and Cumulative Index to Nursing and Allied Health Literature (CINAHL), will be searched for articles of relevance. Further manual searching of reference lists in identified articles will be undertaken to include other studies of relevance. We will also search relevant grey literature databases such as Open Grey and Open Access Theses and Dissertations (OATD) to identify relevant studies.

***Approach to developing search strategy*.** Different sources (e.g. MeSH headings and thesaurus) will be used to identify terms and synonyms to comprehensively cover the research questions as much as possible ^70– 73^. The proposed search strategy was developed in consultation with an academic librarian ( Table 2) for MEDLINE using MesH terms such as "Coronary stenosis", "Carotid Stenosis", "Psychological Distress" and "Quality of Life". We also used Boolean operators “AND” to narrow search results to include only relevant results containing required keywords and “OR” to expand search results and combine synonyms. Other keywords such as behaviour, lifestyle, motivation, risk perception, medication adherence and smoking cessation were included to capture all relevant studies as mental health and HRQoL outcomes are unlikely to be primary or secondary outcomes and thus reported in the title or abstract. This search strategy will be modified for use in other databases. Due to the exploratory nature of scoping reviews and the need to ensure a comprehensive search of relevant literature, an iterative approach to search strategies will be employed ^64^. This implies that the search strategy will be updated as we discover new terms as we work through the review.

**Table 2.** Proposed search strategy. MeSH, Medical Subject Headings; CAC, Coronary Artery Calcium.

"coronary stenosis"[MeSH Terms] OR Coronary Stenosis [Text Word] OR Coronary artery stenosis OR "Carotid Stenosis"[MeSH] OR Carotid plaques OR Carotid ultrasound OR Coronary artery calc* OR Coronary calc* OR CAC score* OR Coronary artery calcium score OR Calcium score
AND
Mental* OR "Psychological Distress"[MeSH] OR Psych* OR "Quality of Life"[MeSH] OR "Anxiety"[MeSH] OR Anx* OR "Depression"[MeSH] OR Dep* OR mood OR Worr* OR alarm OR Lifestyle change OR Behaviour change OR Behaviour OR Lifestyle OR Motivation OR Risk perception OR Risk perception* OR Medication adherence OR smoking cessation

These terms will be searched as keywords in the title and abstract headings and no date limits will be applied. Search results will be downloaded, imported and saved as Microsoft Word and PDF documents. Database outputs will be compared to check for the existence of any duplicates.

### Study selection

**Figure 1.**
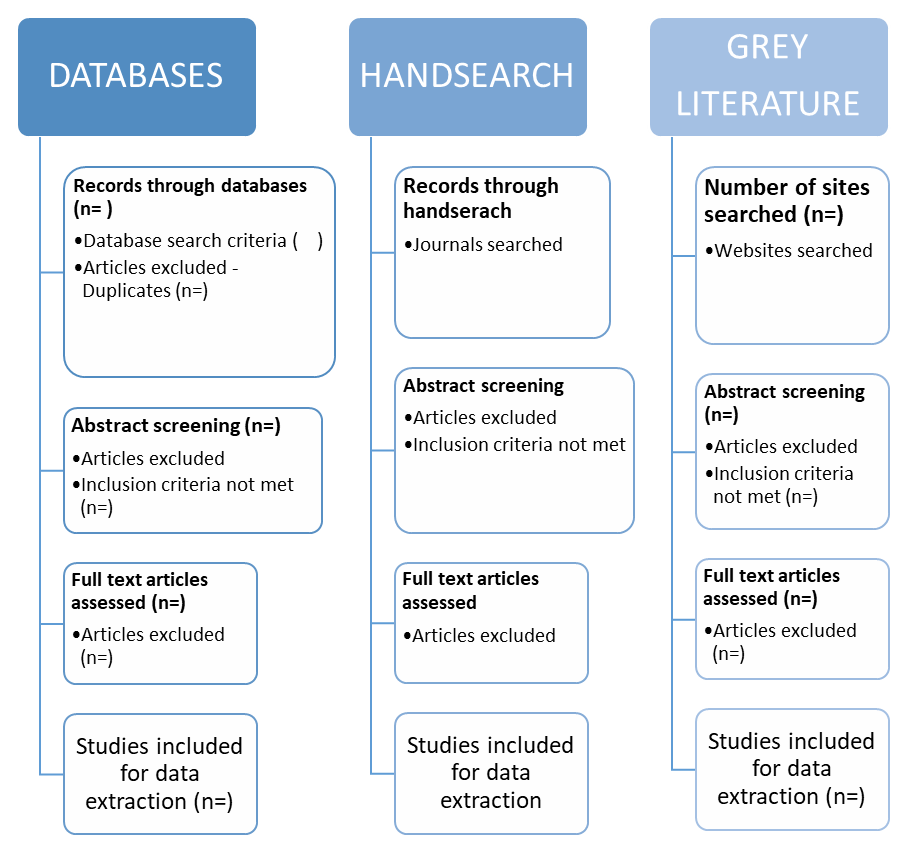
Selection process chart.

Databases and records will be screened using the eligibility criteria (see below) and studies not meeting the criteria will be excluded. The process for identification, screening, eligibility and studies to be included is displayed in Figure 1. The process of searching and selection will be reported in the main review using a PRISMA flowchart ^74^.

The screening will begin with title and abstract screening by two investigators (RA and JRL) who will independently screen the titles and abstracts for all retrieved records for inclusion and to agree on exclusions. This process will be piloted using a sample of abstracts to ensure that this approach will be efficient enough to capture all relevant articles. Any articles that meet the inclusion criteria or that cannot be excluded will be retained for full text review. For the second stage, two investigators (RA and JRL) will each independently screen the full text of articles to determine if they meet the inclusion criteria and conflicts will be resolved by an independent reviewer (LCB) and data from included studies will be extracted.

***Inclusion criteria***

The following inclusion criteria will apply:

***a) Study characteristics***

Studies must be of adults who are 18 years and over and asymptomatic (not screened due to clinical symptoms such as chest pain or angina) and without pre-existing CVD (e.g. stroke, myocardial infarction, peripheral arterial disease or transient ischemic attack). Studies may report follow-up assessment and outcomes such as psychological distress and/or HRQoL after participants received information related to their own coronary artery calcification or carotid stenosis/plaque.

***b) Study types***

Study types that will be included for this scoping review are empirical studies of any type. No year of publication and language restrictions will be applied.

***Concepts***

***i) Imaging results***

Information regarding the state of arteries, extent of stenosis, extent of coronary artery calcification, or carotid/atherosclerotic plaques, coronary calcium score, arterial wall irregularities or obstructive artery walls conveyed to study participants.

***ii) Psychological distress and HRQoL***

An article may report psychological distress (e.g., anxiety, depression, impulsivity, worry, psychoticism, impulsivity, aggression, obsession-compulsion, or interpersonal sensitivity) and/or QoL/HRQoL (i.e. an individual’s self-perceived health status) as an outcome or include QoL/HRQoL measure using a standard instrument to be included in this review.

***c) Context***

This scoping review will include studies conducted in any geographical location among any racial/ethnic group and gender. Studies will be included irrespective of their settings.

***Exclusion criteria***

***a) Study types, participants, and imaging methods***

Studies in symptomatic patients undergoing invasive imaging for diagnostic purposes will be excluded. Other studies that will be excluded are studies providing imaging results of other vascular diseases/conditions such as Aneurysm or Endoleak; Angiodysplasia; Angioedema; Angiomatosis (Bacillary Angiomatosis, Klippel-Trenaunay-Weber Syndrome, Sturge-Weber Syndrome, von Hippel-Lindau Disease); Arteriovenous Malformations; Capillary Leak Syndrome; Ischemic Colitis; Compartment Syndromes; Diabetic Angiopathies; Hand-Arm Vibration Syndrome; Hemorrhoids; Hemostatic Disorders; Hyperemia; Hepatic Veno-Occlusive Disease; Hypotension; Peliosis Hepatis; Ischemic Optic Neuropathy; Pulmonary Veno-Occlusive Disease; Scimitar Syndrome; Retinal Vein Occlusion;Pulmonary Vein Stenosis; Splenic Infarction; Superior Vena Cava Syndrome; Telangiectasis; Varicocele; Thoracic Outlet Syndrome; Varicose Veins; Vascular Fistula; Vascular Neoplasms; Vascular System Injuries; Vasculitis as well as Vasoplegia and Venous Insufficiency.

***b) Outcomes***

Studies without outcomes considered as psychological distress and/or QoL/HRQoL will be excluded. We will also exclude studies where psychological distress/psychiatric and/or QoL/HRQoL assessments were performed only before vascular imaging procedure and not after provision of imaging results.

### Charting the data

A draft data extraction chart will be developed and piloted with a selection of identified studies. The diagrammatic or tabular form of presentation or charting will be used for this study. The potential chart categories may consist of authors information (names, year of publication, study location), participant characteristics (age, gender), research design, methods, instruments/techniques/clinical assessments used to gather data on coronary artery calcification, carotid plaque/stenosis, psychological distress, HRQoL and aims/purpose of the extracted studies ( Table 3). We will also extract data on how vascular imaging results were provided and whether there was additional counselling or support mechanisms.

**Table 3.** Summary of data extraction items. HRQoL, health-related quality of life

RECORD DETAILS	Last name of first author, publication year, journal
STUDY	Study purpose
SETTING	Study location
POPULATION	Age of participants, gender of participants, sample
INTERVENTION	Imaging technique used, results provision details, follow-up period after baseline screening, mental distress and QOL outcome assessment instruments, counselling/additional support for study participants
STUDY DESIGN/ TYPE	As reported by authors or as defined by review team
OUTCOMES	Key psychological distress and/or HRQoL outcomes reported by authors

EndNote X9 will be used as a reference management tool and to avoid duplications. Microsoft Excel and Word will be used to manage data within the review team.

### Collating, summarizing and reporting the results

This review will employ thematic and numeric approaches to summarise studies. A thematic approach will be used to summarise the main and sub-themes that will emerge after the scoping exercise. A numeric approach will also be used to summarise results of the scoping review by presenting the quantity of each emerging concept (e.g. worry was used interchangeably with anxiety (n=2) or most of the studies (n=25) measured depression using the Center for Epidemiological Studies Depression (C-ESD) instrument). The scoping review will not produce a pooled estimate of the impact of vascular imaging results on psychological distress and/or HRQoL as we aim to preliminary assess the potential size, scope and gaps in available literature.

Results on the state of scientific literature will be reported and the gaps in the literature will be identified. There will be further discussion on the implications of the results for practice and future research.

### Study findings and dissemination

The findings from this review will be submitted to peer-reviewed journals to be considered for publication and may be presented at scientific conferences. Also, we aim to share our results with key stakeholders to influence policy and practice.

### Quality appraisal

Several authors ^65, 75, 76^ criticised Arksey and O’Malley ^64^ for originally suggesting that quality assessment is not necessary for scoping reviews and reported it as a limitation. We will therefore employ a risk of bias assessment to highlight the strengths and weaknesses of the included studies to inform the interpretation of the evidence ^77^. We will use the ROBINS-I tool for assessing risk of bias in non-randomised control trials to grade the quality of evidence. Included studies will be described as low risk of bias, moderate risk of bias, serious risk of bias, or critical risk of bias ^78^. Randomised controlled trials will be assessed for risk of bias using the revised Cochrane risk-of-bias tool for randomized trials (RoB 2) ^79^. Findings will be graded as low, high or with some concerns.

## Study status

Start date of search: August 2020; anticipated date of completing review: July, 2021

***Current study status:***

Preliminary searches: Yes

Piloting search strategy: Yes

Pilot screening of search results: Yes

Study selection process piloting: Yes

Formal screening of search results against eligibility criteria: Started

Data extraction: Not started

Data analysis and interpretation: Not started

## Conclusion

The purpose of this protocol is to describe the methodological considerations that will guide the completion of a scoping review that will summarise the extent, range and nature of studies on psychological distress and/or HRQoL outcomes reported among asymptomatic adults following the provision of vascular imaging results. This comprehensive review will help advance knowledge about potential negative effects of screening for asymptomatic CVD to elicit healthful behaviour changes. It could also possibly enable the development of strategies to prevent distress. The results of this review will help advance knowledge in this field and will be useful for future medical practice when providing vascular imaging results to patients, cardiovascular research, and future clinical trials providing vascular imaging results to participants. This scoping review will be limited to studies reporting coronary or carotid artery plaque screening only as these are the commonly used structural vascular imaging modalities for large screening initiatives of asymptomatic individuals.

## Ethics approval and consent to participate

There will be no formal ethical application and ethical review as no primary data will be collected.

## Data availability

No data are associated with this article.
