# Supplementary material for: Psychological distress and quality of life following provision of vascular imaging results of the coronary and carotid arteries to asymptomatic adults: a scoping review protocol
Source: F1000Res. 2021 Nov 9;9:1376. Originally published 2020 Nov 26. [Version 3] doi: 10.12688/f1000research.27432.3 (PMC8733827; doi:10.12688/f1000research.27432.3)
Supplement: Peer review history

# Peer review: Reviewer response for version 3

**DOI:** 10.5256/f1000research.78990.r99576

**Published:** 2022-01-05

**Authors:** Aaron Conway

**Affiliations:** 1 Peter Munk Cardiac Centre, Toronto, Canada

The authors have addressed my comments from the initial version.

Is the study design appropriate for the research question?

Yes

Is the rationale for, and objectives of, the study clearly described?

Yes

Are sufficient details of the methods provided to allow replication by others?

Yes

Are the datasets clearly presented in a useable and accessible format?

Not applicable

Reviewer Expertise:

I have published many systematic reviews in the area of cardiovascular care.

I confirm that I have read this submission and believe that I have an appropriate level of expertise to confirm that it is of an acceptable scientific standard.

# Peer review: Reviewer response for version 2

**DOI:** 10.5256/f1000research.56954.r93685

**Published:** 2021-10-06

**Authors:** Aaron Conway

**Affiliations:** 1 Peter Munk Cardiac Centre, Toronto, Canada

This protocol for a scoping review is well-written and provides adequate detail about the proposed methods. In general, the methods seem well-justified given the research design. My one suggestion is to re-consider the intended use of Cochrane risk of bias assessment tools. Most up to date guidance for scoping reviews include recommendations that critical appraisal and risk of bias assessment is not consistent with the aims of this systematic review design. For example: Peters *et al.* (2020 ^1^).

Is the study design appropriate for the research question?

Yes

Is the rationale for, and objectives of, the study clearly described?

Yes

Are sufficient details of the methods provided to allow replication by others?

Yes

Are the datasets clearly presented in a useable and accessible format?

Not applicable

Reviewer Expertise:

I have published many systematic reviews in the area of cardiovascular care.

I confirm that I have read this submission and believe that I have an appropriate level of expertise to confirm that it is of an acceptable scientific standard, however I have significant reservations, as outlined above.

## References

[1] : Updated methodological guidance for the conduct of scoping reviews. *JBI Evid Synth* .18(10) : 10.11124/JBIES-20-00167 2119-2126 10.11124/JBIES-20-00167 33038124

# Response

**Published:** 2021-11-01

**Authors:** Reindolf Anokye

**Affiliations:** Edith Cowan University, Australia

We would like to thank the reviewer for his time reviewing the manuscript. We have considered the suggestion, other recommendations such as *PRISMA* extension for scoping reviews (2018), and the outcomes that will be reported in this scoping review and have decided not to include any critical appraisal and risk of bias assessment for this review.

# Peer review: Reviewer response for version 2

**DOI:** 10.5256/f1000research.56954.r86346

**Published:** 2021-07-16

**Authors:** Md Mahbub Hossain, Abida Sultana, Samia Tasnim

**Affiliations:** 1 Department of Health Promotion and Community Health Sciences, School of Public Health, Texas A&M University, College Station, USA; 2 Gazi Medical College, Khulna, Bangladesh

The updated version of the protocol addressed all major concerns that were mentioned in the prior peer-review comments.

Is the study design appropriate for the research question?

Partly

Is the rationale for, and objectives of, the study clearly described?

Yes

Are sufficient details of the methods provided to allow replication by others?

Partly

Are the datasets clearly presented in a useable and accessible format?

Not applicable

Reviewer Expertise:

Psychosocial Epidemiology; Evidence-based Mental Health; Mental Health Policies and Systems

We confirm that we have read this submission and believe that we have an appropriate level of expertise to confirm that it is of an acceptable scientific standard.

# Peer review: Reviewer response for version 1

**DOI:** 10.5256/f1000research.30316.r77059

**Published:** 2021-03-08

**Authors:** Md Mahbub Hossain, Abida Sultana, Samia Tasnim

**Affiliations:** 1 Department of Health Promotion and Community Health Sciences, School of Public Health, Texas A&M University, College Station, USA; 2 Gazi Medical College, Khulna, Bangladesh

We want to express our gratitude to the authors who have chosen to work on such an important area of health science. With the growing burden of cardiovascular diseases, it is necessary to understand how such diagnoses may impact mental health and overall wellbeing in affected individuals. This prospective scoping review is likely to add great value; however, there are a few concerns that require to be addressed before the review protocol is indexed, and more importantly before the review is conducted.

 First, the authors planned to use the framework by Arksey and O'Malley, which is one of the most widely used frameworks for scoping reviews. However, this framework has undergone further improvements by Levac et al. (2010) ^1^ and Peters et al. (2015, 2017) ^2^^,^^3^^,^^4^. The authors may consider using the updated frameworks or give the rationale for using the current one over the more recent versions.

 Second, in Table 1, the authors mentioned adults and international within core elements such as "perspective" and "setting," respectively. A researcher and/or a practitioner may wish to know where the population belonged to in the primary studies, which can be local/global as well as community/clinical settings. I'd suggest using "population" for adults and expanding the concept of "setting" to the community and/or clinical settings while keeping the search terms and the scope of the review as global. This would bring more clarity and might make more sense from a systematic assessment perspective on the evidence.

 Third, the authors must explain what "mental distress" and "quality of life" are. These concepts have varying definitions from different disciplines. It may not be feasible to do another review to summarize what they mean; however, it would be useful to have at least a working definition of these concepts that refers to some of the leading articles explaining these terms. Such explanations would be helpful to present and discuss the findings of the review in the future. However, the protocol must mention these clearly before the review begins.

 Lastly, in the concepts section, the authors mentioned that "studies must report" mental distress and quality of life. The use of "must" in both concepts creates a dilemma that is they will recruit articles if they (must) include both these concepts. We found this idea less practical. Rather, an article may report either "mental distress" or "quality of life," and the authors may present both as the summarized evidence, which would provide a better "map" of the evidence landscape. We would humbly request the authors to make necessary changes that reflect the true objective of the review, as they feel appropriate.

Is the study design appropriate for the research question?

Partly

Is the rationale for, and objectives of, the study clearly described?

Yes

Are sufficient details of the methods provided to allow replication by others?

Partly

Are the datasets clearly presented in a useable and accessible format?

Not applicable

Reviewer Expertise:

Psychosocial Epidemiology; Evidence-based Mental Health; Mental Health Policies and Systems

We confirm that we have read this submission and believe that we have an appropriate level of expertise to state that we do not consider it to be of an acceptable scientific standard, for reasons outlined above.

## References

[1] : Scoping studies: advancing the methodology. *Implement Sci* .2010;5: 10.1186/1748-5908-5-69 69 10.1186/1748-5908-5-69 20854677PMC2954944

[2] : Guidance for conducting systematic scoping reviews. *Int J Evid Based Healthc* .2015;13(3) : 10.1097/XEB.0000000000000050 141-6 10.1097/XEB.0000000000000050 26134548

[3] : In no uncertain terms: the importance of a defined objective in scoping reviews. *JBI Database of Systematic Reviews and Implementation Reports* .2016;14(2) : 10.11124/jbisrir-2016-2838 1-4 10.11124/jbisrir-2016-2838 27536788

[4] : Chapter 11: Scoping Reviews.2020; 10.46658/JBIMES-20-12 10.46658/JBIMES-20-12

# Response

**Published:** 2021-05-17

**Authors:** Reindolf Anokye

**Affiliations:** Edith Cowan University, Australia

*We want to express our gratitude to the authors who have chosen to work on such an important area of health science. With the growing burden of cardiovascular diseases, it is necessary to understand how such diagnoses may impact mental health and overall wellbeing in affected individuals. This prospective scoping review is likely to add great value; however, there are a few concerns that require to be addressed before the review protocol is indexed, and more importantly before the review is conducted.*

**Response:**

 We would like to thank the reviewers for their time and valuable comments when reviewing the manuscript. We have extensively revised the manuscript to address the comments and feedback.

*First, the authors planned to use the framework by Arksey and O'Malley, which is one of the most widely used frameworks for scoping reviews. However, this framework has undergone further improvements by Levac et al. (2010) and Peters et al. (2015, 2017). The authors may consider using the updated frameworks or give the rationale for using the current one over the more recent versions.*

**Response:**

 We agree that the updated framework would better suit this study and provide a better approach to the design of the study including collating, summarizing and reporting the results. The framework has been updated in the manuscript. Please see the study design section for details of the updated framework.                                                                                                   

*Second, in Table 1, the authors mentioned adults and international within core elements such as "perspective" and "setting," respectively. A researcher and/or a practitioner may wish to know where the population belonged to in the primary studies, which can be local/global as well as community/clinical settings. I'd suggest using "population" for adults and expanding the concept of "setting" to the community and/or clinical settings while keeping the search terms and the scope of the review as global. This would bring more clarity and might make more sense from a systematic assessment perspective on the evidence.*

**Response:**

 Table 1 has been updated as per comments under ‘study rationale and guiding question’ section of the manuscript. We have now expanded the concept of “setting” to include community and/or clinical settings and also using “population” for adults while keeping the scope of the review global.

*Third, the authors must explain what "mental distress" and "quality of life" are. These concepts have varying definitions from different disciplines. It may not be feasible to do another review to summarize what they mean; however, it would be useful to have at least a working definition of these concepts that refers to some of the leading articles explaining these terms. Such explanations would be helpful to present and discuss the findings of the review in the future. However, the protocol must mention these clearly before the review begins.*

**Response:**

 We agree that "mental distress" and "quality of life" could have been explained better in the manuscript. We have replaced mental distress with psychological distress in the manuscript even though articles using mental distress will still be included in the review. Although mental distress and psychological distress are often used interchangeably, psychological distress is well defined in the literature and is perhaps more easily understood. We have also expanded our definition of quality of life and indicated that our focus is on health-related quality of life. The explanation or definition for psychological distress and health-related quality of life can be found in the third paragraph of the introduction section of the manuscript.

*Lastly, in the concepts section, the authors mentioned that "studies must report" mental distress and quality of life. The use of "must" in both concepts creates a dilemma that is they will recruit articles if they (must) include both these concepts. We found this idea less practical. Rather, an article may report either "mental distress" or "quality of life," and the authors may present both as the summarized evidence, which would provide a better "map" of the evidence landscape. We would humbly request the authors to make necessary changes that reflect the true objective of the review, as they feel appropriate.*

**Response:**

 We have replaced "studies must report" with “an article may report” psychological distress and/or quality of life/health-related quality of life for inclusion in the review. This can be found in the concepts section of the manuscript.
